# Review of applications of high-throughput sequencing in personalized medicine: barriers and facilitators of future progress in research and clinical application

**DOI:** 10.1093/bib/bby051

**Published:** 2019-06-14

**Authors:** Gaye Lightbody, Valeriia Haberland, Fiona Browne, Laura Taggart, Huiru Zheng, Eileen Parkes, Jaine K Blayney

**Affiliations:** 1 School of Computing, Ulster University, Newtownabbey, UK; 2 MRC Integrative Epidemiology Unit, Population Health Sciences, Bristol Medical School, University of Bristol, Bristol, UK; 3 Almac Diagnostics, Craigavon, UK; 4 Centre for Cancer Research & Cell Biology, School of Medicine, Dentistry and Biomedical Sciences, Queen's University, Belfast, UK

**Keywords:** high-throughput sequencing, personalized medicine, clinical translation, translational research, high-performance computing, grid computing, cloud computing

## Abstract

There has been an exponential growth in the performance and output of sequencing technologies (omics data) with full genome sequencing now producing gigabases of reads on a daily basis. These data may hold the promise of personalized medicine, leading to routinely available sequencing tests that can guide patient treatment decisions. In the era of high-throughput sequencing (HTS), computational considerations, data governance and clinical translation are the greatest rate-limiting steps. To ensure that the analysis, management and interpretation of such extensive omics data is exploited to its full potential, key factors, including sample sourcing, technology selection and computational expertise and resources, need to be considered, leading to an integrated set of high-performance tools and systems. This article provides an up-to-date overview of the evolution of HTS and the accompanying tools, infrastructure and data management approaches that are emerging in this space, which, if used within in a multidisciplinary context, may ultimately facilitate the development of personalized medicine.

## Introduction

Over the past decade, there have been exponential advances in our capacity to sequence a human genome. As recently as 2016, it would have taken over a day [1]. Now, using current technology [2], it is possible to process a genome sequence within an hour [3, 4]. The development of high-throughput sequencing (HTS) technologies has been central to achieving this, with massively parallel sequencing offering larger throughput than the conventional Sanger sequencing [5] approach.

While advances have been made across all aspects of the sequencing workflow, the focus on platform development has made a significant contribution to driving down machine size and HTS costs while facilitating performance gains. In addition, this has been enhanced by reductions in both the cost of computational power and size, as expected through Moore’s law [6]. However, since 2007 [7], the reduction in the sequencing cost per genome has surpassed Moore’s law; thus, we are now in the era of the sub-$1000 genome. An extensive review of the past 10 years of HTS can be found in [8] along with additional technological solutions in [9, 10] and more recently in [11].

The decreasing costs of HTS have brought it within the reach of smaller laboratories, facilitating the generation of high-dimensional in-house data sets, with typical HTS devices producing over 100 gigabases (Gb) of reads in 24 h [12]. As with other examples of ‘Big Data’, the steps involved in the design, pre-processing, normalization and downstream analysis of HTS data are significant. Furthermore, there are substantial challenges presented, including sample collection and quality control, selection of HTS technology, to the integration of data sets across platforms and technologies. HTS data therefore present its own set of *in silico* and computational challenges, leading to a ‘Data Deluge’ [13] in which the emphasis has moved from data generation to the ability to store, access, share and analyse the data effectively. As reported by Sboner *et al.* [14], these additional elements contribute towards a more realistic assessment of the true cost of HTS use. In addition, there are also data governance and patient privacy implications, particularly resulting from the speed of change brought about by the application of HTS in clinical workflows [15, 16].

Considering these intersecting challenges within the biomedical domain, particularly with regard to clinical (and commercial) translation, HTS can be considered from the perspectives of four key stakeholders: biologists, clinicians and patients alongside bioinformaticians/computer scientists. Against this background, we consider common HTS bottlenecks that can be encountered at different workflow stages. We then present potential *in silico* and computational solutions, extending on the review in [17], and examine further rate-limiting issues that may in turn be raised. We therefore conclude with a discussion on the future role of HTS in facilitating biomedical research and its potential translation to clinical decision-making tools.

## HTS: from biomedical research to clinical application

In the biomedical domain, HTS can be used to characterize biological markers (biomarkers), including genes and proteins, often derived from human tissue or blood, to understand disease development and progression and/or predict treatment response or patient survival [18]. Biomarkers can be classified into three categories: diagnostic (presence or absence of disease), predictive (how a patient responds to treatment) and prognostic (how long a patient survives post-intervention) [18].

**Figure 1.**
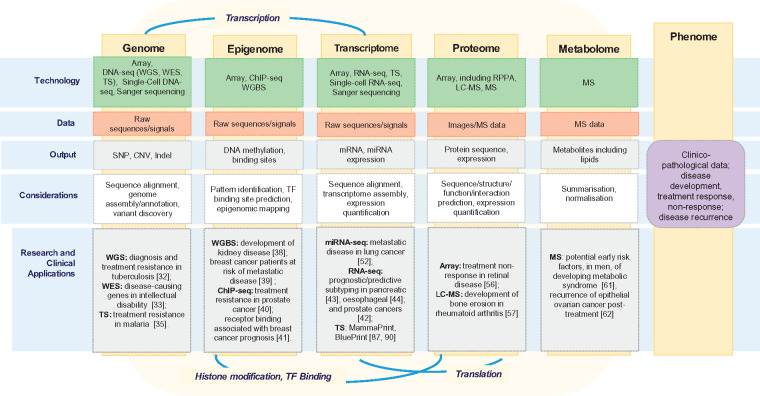
Summary schema of omics levels with associated technologies, data types, outputs, analytical considerations and research and clinical applications. Five levels or components, genome, epigenome, transcriptome, proteome and metabolome are presented, all of which can be considered with respect to the phenome (common patient characteristics). Key associations between omics levels are also represented, including transcription (between the genome and transcriptome), histone modification and TF-binding (connecting the epigenome with the proteome) and translation (from the transcriptome to the proteome). *Source:* Adapted from: [19–21] and [22].

Markers and drivers of disease development, progression and treatment response can be detected at the deoxyribonucleic acid (DNA), ribonucleic acid (RNA) or protein levels with a range of HTS techniques (Figure 1). We consider both biomarker and HTS applications at five key omics levels, genome, epigenome, transcriptome, proteome and metabolome. These levels are connected via genetic data transfer processes, including transcription, translation, binding and protein modification [23, 24]. As shown in Figure 1, each of these omics levels can be considered with respect to patient characteristics, such as risk of disease or response to a treatment, i.e. phenotypes.

At the genome (DNA) level, alterations in genes are analysed, e.g. single-nucleotide polymorphisms (SNPs), indels, copy number variations (CNVs) and fusion genes [25] (Figure 1). SNPs (equivalent to ‘typos’ in the genome), indels (insertion or deletion of bases in a sequence) and CNVs (where portions of the genome are repeated) have been linked to disease susceptibility. SNPs in the *VCAM-1* and *ARFGEF2* genes, a deletion in the *CTFR* gene and CNVs in the *HLA* gene were found to be associated with cases of sickle cell anaemia [26], cystic fibrosis (CF) [27] and rheumatoid arthritis [28], respectively. Fusion genes, when two individual genes form a hybrid gene, can also be associated with disease development, as with *TMPRSS2-ERG* in prostate cancer [29]. Microarrays [30], and more recently, DNA-sequencing (DNA-seq), comprising whole-genome sequencing (WGS), whole-exome sequencing (WES) and targeted sequencing (TS) have been used to study these alterations. WGS enables the interrogation of alterations in both the coding and non-coding regions of the genome [31] and has been used to identify multiple SNPs relating to the diagnosis of tuberculosis and treatment resistance [32]. WES is limited to coding regions, approximately 1% of the genome [31]. A more affordable option than WGS, WES may omit potentially informative gene regulation regions, though its use was well founded in a study of intellectual disability, as three novel disease-causing candidate genes were identified [33]. TS focuses on specific regions of the genome and is useful when prior information is known about the disease [34], e.g. in a study to understand resistance to first-line antimalarial therapy, TS identified six novel resistance-causing mutations [35].

Epigenomics encompasses the chemical modification, through internal or external factors, of DNA, which in turn can repress the corresponding gene expression, leading to disease or treatment resistance (Figure 1) [36]. Both microarray and sequencing technologies can be used to quantify DNA methylation status. Bisulphite conversion is necessary for both older (microarray) and newer sequencing-based technologies, facilitating the detection of methylated cytosines (one of four DNA component bases), though is a harsh process that can affect the quality of DNA for downstream analysis [37]. Whole-genome bisulphite sequencing (WGBS) was used to identify methylation of the *IFITM3* gene as a candidate in the development of kidney disease [38]. Legendre *et al.* [39] used WGBS to develop a blood-based methylation patterns that could be used to stratify breast cancer patients into metastatic disease risk groups. Chromatin immunoprecipitation sequencing (ChIP-seq) allows for the precise characterization of transcription factor (TF)-binding sites (location at which a protein binds to DNA to initiate transcription) and patterns of histone (a DNA packaging protein) modification, both of which can affect gene expression. Using this technology, advances in understanding the impact of the epigenome on the development of metastatic disease in patients with early prostate cancer were made [40]. Within oestrogen receptor-positive (ER+) breast cancer, the use of ChIP-Seq helped to identify the prognostic role of the gene *FOXA1* in facilitating ER-binding [41].

The transcriptome encompasses all RNA found in the cell (Figure 1). Messenger RNA (mRNA) is the most commonly studied form of RNA. The transcriptome, capturing the downstream signals from the genome and epigenome, has been used for molecular subtyping and studying drug response [42–45], applying both microarray and HTS technologies. For example, using microarray technology, four breast cancer subtypes associated with patient response to chemotherapy were defined based on a set of RNA patterns (PAM50) [46]. Other RNA types such non-coding RNAs and microRNA (miRNA) have also been described [47]. In particular, miRNA has been shown to be important in disease development and progression through gene regulatory functionality [48]. miRNAs have been associated with relapsing–remitting multiple sclerosis [49] and dormancy of the human immunodeficiency virus type 1 (HIV-1) in patients treated with antiretroviral therapy [50]. RNA can be studied through both microarray and RNA-Sequencing (RNA-Seq) with RNA-Seq also allowing for the discovery of additional modifications, e.g. fusion genes, similar to the genome level [51]. RNA-Seq has also been extensively used, often within a multi-omics or integrative context. This has resulted in the characterization of novel molecular subgroups associated with treatment response and/or survival in multiple cancer studies, including pancreatic [43], oesophageal [44], prostate [42] and cholangiocarcinoma [45]. microRNA sequencing (miRNA-Seq) has also been used in the identification of miRNAs that were significantly associated with remote metastatic disease in lung adenocarcinoma [52].

All the previous elements (genome, epigenome and transcriptome) contribute to the proteome, the set of proteins that comprise an organism (Figure 1) [53]. The sequence, structure and expression of proteins are encoded by the genome but can be altered at the transcriptional level with the potential for changes being introduced at translation [53]. In comparison with other omics levels, it is relatively poorly characterized [54]. Array-based methods, including reverse phase protein array (RPPA) and mass spectrometry (MS) technologies can be applied at this level [55]. Using an array-based technology, Velez *et al.* [56] identified protein targets for a tailored treatment of a patient with inflammatory disease of the retina, reversing sight loss. In addition to array-based methods, MS or liquid chromatography (LC)-MS can be used to study the sequence and structure of proteins, each having a unique weight (mass) fingerprint that can be used to identify their presence in a sample [55]. Liao *et al.* [57] used LC-MS to identify candidate proteins in samples obtained from rheumatoid arthritis patients with none erosion.

Metabolomics is the study of the chemical fingerprints that cellular processes leave behind (Figure 1), i.e. metabolites, which are small molecules, such as amino acids or lipids, resulting from the breakdown of proteins through protein–protein interactions [19]. Similar to proteins, metabolites are identified and studied by MS generating metabolite profiles. The study of metabolites is a well-established and important element in drug discovery, particularly the understanding of the metabolism of a drug and potential associated toxicities [58]. Lipidomics, the study of lipid levels, such as cholesterol and triglyceride, in blood and tissue is a fast-emerging sub-field within metabolomics [59, 60]. Using MS, Sales *et al.* [61] characterised a ‘lipotype’ in men that corresponded to a potential risk of developing metabolic syndrome. While, Ke *et al.* [62] discovered that in epithelial ovarian cancer, patients post-surgery, who had recurred were found to have high levels of lipid and amino acid metabolism.

### Research applications of HTS

The research community’s reliance on microarray technology is now being replaced by a welcoming endorsement of sequencing technologies. This trend can be seen in the work of the flagship The Cancer Genome Atlas (TCGA) consortium [63]. In 2008, the first TCGA publication in glioblastoma used Sanger sequencing and array-based technologies to analyse patient samples at the genomic, epigenomic and transcriptomic levels [64]. In 2017, WES, RNA-Seq and miRNA-Seq, in addition to array-based SNP, methylation and protein analysis were used in a study of uterine carcinosarcoma [65]. As predicted [66], sequencing, particularly, RNA-Seq technology is rapidly replacing microarray-based approaches, because of its technical superiority [67] and ability to derive novel biological insights [68]. Moreover, using data from TCGA, pan-cancer studies analysing data from 10 000 solid tumours identified the impact of important biologies such as impaired DNA damage response [69] and comprehensive immune biology across cancers [70]. Indeed, this subsequent improvement in understanding the driving biologies and potential vulnerabilities of cancers demonstrate the importance of HTS in advancing our understanding of disease.

### Clinical applications and limitations of HTS

HTS has also been applied within clinical trial contexts including in the development of early cancer detection assays or tests and selection of new treatments for patients not responding to standard regimes [71, 72]. Three current clinical trials use TS at the genome level (Figure 1). The first, the STRIVE Study, is using sequencing to detect and analyse circulating cell-free nucleic acids, present in blood samples taken from patients who had undergone a screening mammogram to improve early detection of breast cancer [73]. Another trial, NCI-Match, has enrolled cancer patients (with solid tumours or lymphomas) that had received treatment, yet had progressed, to help determine drug repurposing options, thereby improving outcomes for cancer patients [74]. Another exploratory study, the Michigan Oncology Sequencing Project (Mi-ONCOSEQ) uses a multi-sequencing approach to stratify clinical trial-eligible patients, with metastatic or refractory cancers. Mi-ONCOSEQ also considers the bioethical issues surrounding genomic testing and results disclosure to patients and clinicians [75].

Reaching the clinical trial stage does not always result in success. Despite identifying a drug–target mutation in nearly half of patients enrolled, the MOSCATO trial [76] reported the ability to deliver this therapy in less than one quarter of patients, of whom 11% responded. The large numbers screened for a limited clinical response is an important shortcoming of current HTS approaches. Despite this, HTS approaches have already resulted in improved outcomes. Sequencing the genome of one exceptional responder in a failed clinical trial of everolimus in bladder cancer, an inhibitor of the gene *mTOR*, identified a mutation of a key *mTOR* regulator, the *TSC1* gene [77]. Further sequencing discovered this mutation in 8% of bladder cancers. Initiatives are now ongoing to sequence exceptional responders in clinical trials to identify other, currently unknown, targetable mutations [78], demonstrating the prospective potent impact of HTS.

Although HTS-based clinical trials may not always fulfil their original potential, crucially, platform and diagnostic acceptance of HTS by regulatory bodies has been forthcoming. In 2013, the Illumina MiSeqDx was the first HTS platform to be approved as an *in vitro* diagnostic tool by the Food and Drug Administration (FDA), alongside two Illumina diagnostic assays, the CF Clinical Sequencing and CF 139-Variant assays, both of which target the region around the *CFTR* gene at the genomic level, for screening and diagnosis purposes [79–81]. Later, in 2016, the FDA published draft guidance for the development of further HTS-based assays for rare inherited diseases [82]. Then, in 2017, the FDA approved a further three HTS-based *in vitro* diagnostic tests, including FoundationOne’s companion diagnostic, F1CDx [83], Memorial Sloan Kettering Cancer Center’s MSK-IMPACT [84] and Thermo Fisher Scientific’s Oncomine Dx Target Test [85, 86]. Both F1CDx and MSK-IMPACT can detect sequence modifications in various cancers to identify patients who may benefit from a number of targeted therapies [83, 84]. Similarly, Thermo Fisher Scientific’s Oncomine Dx Target Test also quantifies genomic sequence changes in tumours to guide treatment for non-small cell lung cancer [86].

### Translating research into clinical applications

However, regulatory approval does not equate to a global clinical acceptance and uptake. A number of breast cancer predictive transcriptome-based tests were derived in the pre-HTS era, such as PAM50 (Prosigna, NanoString Technologies, United States) [46, 87] and MammaPrint (MammaPrint BluePrint, Agendia BV, The Netherlands) [88, 89], both of which were later developed into commercial tests, the latter using RNA-Seq. Both were approved by both the FDA for use in the United States and in the European Economic Area through the Conformité Européene (CE) mark [82, 90–92] and included in the updated clinical decision-making guidelines from the European Group on Tumor Markers [93]. However, the National Comprehensive Cancer Network [94], while acknowledging other tests were available, only referred to the possible use of the OncotypeDx assay (Genomic Health, CA, USA) [95], which was developed using an older, targeted, RNA-quantification technology, reverse transcription polymerase chain reaction. Understandably, there is still a sense of caution regarding the use of HTS in a clinical context [96, 97], with an argument that further randomized trials are required to demonstrate the effectiveness of approved tests.

In choosing an HTS technology, users need to consider not only the biological hypothesis being tested but also sample collection and quality control issues, together with downstream computational and analytical overheads associated with a chosen platform. Whether working at the research or clinical translation level, a multidisciplinary approach is required at each HTS stage, bringing together clinicians, biologists and bioinformaticians to ensure ultimate patient benefit.

## HTS platforms, pipelines and challenges

**Figure 2.**
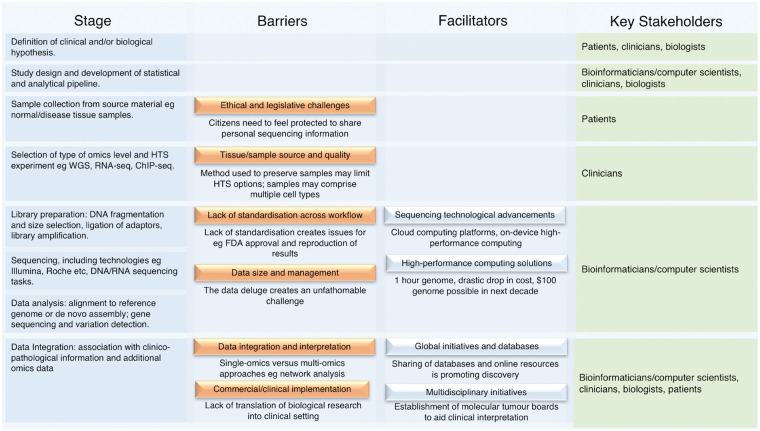
Overview of stages, barriers, facilitators and stakeholders in HTS pipelines from hypothesis setting to clinical interpretation. Eight common stages involved within a generic HTS pipeline/workflow are presented, set against factors acting as barriers to, or facilitators of, progress towards commercial/clinical translation and key stakeholders.

Against this heterogeneous background of regulatory approval and clinical acceptance, we examine additional barriers to and facilitators of HTS application to personalized medicine. We consider the key initial challenges, including sample collection [98] and quality [99], choice of platform [100], library preparation [101] and sequencing and data analysis [100] (Figure 2). We also highlight key stakeholders at each level.

### Sample collection

Patient tissue forms the backbone of personalized medicine research. Samples for analysis may originate from formalin-fixed, paraffin-embedded (FFPE) or fresh-frozen samples. With FFPE, sample quality can be compromised by RNA degradation, leading to HTS library construction failure [98] (Figure 2). Microarray platforms have been developed to reliably quantify transcription from FFPE samples [102]. Although results with respect to RNA-Seq have been promising [103], some suggest that the bottleneck of RNA degradation currently restricts the use of HTS to DNA-seq, e.g. TS or WES [104]. With regard to the latter, the limited concordance between a WES study of fresh-frozen and FFPE melanoma samples raises concerns [105]. Where there is prior knowledge of a disease, a TS approach, focused on selected genes or regions, can be more appropriate, maintaining resolution, with increasing efficiency and affordability [106]. With the development of FFPE-tailored pre-processing pipelines alongside refinement in the underlying technologies, it is expected that HTS accuracy will potentially improve, enabling clinical uptake [105, 107]. An alternative approach to adjusting the technology would be to switch to fresh-frozen tissue (or adopt a combined strategy). Such a move would involve input from clinicians, including surgeons and pathologists, particularly in biobanks. This would represent a much more efficient alternative to FFPE, with less technical limitations and could facilitate faster clinical decision-making, though it can present considerable storage and maintenance implications [108].

### Sample heterogeneity

Once a sample has been taken from tissue, its composition can be affected by heterogeneity, e.g. in tumour samples, signals may originate from multiple cell types including stroma and immune compartments [99] (Figure 2). This composition varies across samples and has implications for biomarker development, with the potential to confound results. At a bioinformatics level, *in silico* optimisation and/or gene list-based approaches have been applied to separate out signals (termed deconvolution) into their respective cell types [99, 109–112]. Once stratified into separate cell-type components, standard downstream analyses can follow. Experimental (biological) alternatives, namely, cell-specific HTS technologies, are also being used. Single-cell RNA-Seq (scRNA-Seq) has been successful in predicting treatment response in lung adenocarcinoma [113], glioblastoma [114] and melanoma [115]. The processing particularly of scRNA-Seq data requires special consideration. Standard methods, as used with ‘bulk’ or multi-cell data, are not always appropriate [116–118]. While scRNA-Seq may appear to be a viable alternative to *in silico* approaches, it has been suggested that cell-sorting or cell isolation experimental methods may in turn alter gene expression levels [119].

### Platform choice

While sample type considerations may impact on platform choice, an overall assessment of an HTS platform’s abilities, relative strengths and weaknesses, from biological, clinical and bioinformatics perspectives, will facilitate the appropriate application of the resultant data [100] (Figure 2). Recent platform examples include the Illumina^®^ MiSeq [120], Ion PGM™ (Personal Genome Machine) [121], the PacBio RS II [122] and Qiagen Gene Reader (Sequencing-By-Synthesis) [123]. Last year, the NovaSeq Series from Illumina exceeded existing performance measures guaranteeing an average sequencing time of 1 h per genome [2]. Genomics England has had a partnership with Ilumina since 2014 [124] and has more recently in 2018 extended its partnerships to include Edico Genomics. This new alliance offers a high-performance DRAGEN Bio-IT Platform [4, 125] that reports performance greater than the 2017 NovaSeq solution. An extensive review of the past 10 years of HTS can be found in [8] along with additional technological solutions in [9, 10] and more recently in [11].

### Library preparation

Once a suitable platform has been selected, library preparation, the conversion of nucleic acid materials derived from tissue, etc., into a form suitable for sequencing input, is the next key but potentially a challenging step [101] with biological and bioinformatics implications (Figure 2). Amplification of libraries by polymerase chain reaction (PCR) is prone to introducing bias; although PCR-free methods exist, these too are not challenge-free [101]. Library preparation methods are crucial when only small amounts of DNA be obtained from clinical samples. Sundaram *et al.* [126] compared seven library preparation methods for ChIP-Seq analysis of HeLa cell lines (a preclinical model of cervical cancer) against a PCR-free library preparation approach. This study concluded that there was an inverse correlation between the number cycles of amplification and performance.

### Sequencing

There are different HTS approaches depending on the choice of platform, each of which uses bespoke protocols. As such, the output from data from different HTS workflows/platforms can vary [127]. This lack of standardization can present a challenge when comparing the quality and accuracy of output (Figure 2). Although primarily a bioinformatics issue, both biologists and clinicians need to be aware of how different protocols can impact results. Within a clinical diagnostic context, accuracy, reproducibility and standardization of HTS results can be improved through focusing on the development of reference standards [128].

Regardless of technology applied, the initial analysis or base-calling (whereby bases are assigned to peaks) is usually performed using platform-associated proprietary software. Alignment to a reference genome, or alternatively *de novo* assembly, is next performed. Novel methods in both sequence alignment and assembly are routinely proposed and published [129], such as the cloud computing-based CloudBurst and Rainbow [130, 131]. Additionally, enabling technologies such as Hadoop MapReduce can be used to implement algorithms, including RMAP and Bowtie [132] (covered in the ‘HPC Solutions’ section).

### Data analysis and interpretation

Post-alignment, the appropriate analysis of data is central to an HTS project [133] (Figure 2). As the size and complexity of HTS data increase, the development of new analytical methods is required, optimization for speed and memory usage being key [9]. Given the relative youth of HTS, the lack of consensus between HTS analytical methodologies is not surprising [128, 134]. Regardless of hypothesis, platform, library preparation, sequencing protocol or downstream analytical algorithm, it is clear that HTS usage will demand extensive use of resources, both technical and human. The recruitment of skilled bioinformaticians, who can develop and manage the most appropriate tools and work within a multidisciplinary context, is crucial. Therefore, training, and standardization of training, in the use of HTS technologies is also key, as recognized by the NGS Trainer Consortium [135, 136].

### Analytical/computational challenges

HTS data sets are both high-dimensional and complex in structure. Integrating such data with other data sets, platforms or technologies, to obtain a complete disease profile, is therefore both algorithmically and computationally challenging. A comprehensive review of meta-omics (integration of independent data sets at the same omics level) and poly-omics (integration of different omics types) algorithmic approaches is presented in Ma and Zhang [22]. Poly-omics projects such as TCGA have applied consensus-based methods to detect connecting patterns between different omics levels, e.g. Cluster of Cluster Assignments (COCA) [137] in breast cancer [138] and iCluster [139] in application to prostate cancer [42] and hepatocellular carcinoma [140]. Alternatively, network-based approaches [141–143] to data analysis have the potential to integrate data from disparate sources, while providing clinically relevant results. Multidisciplinary initiatives such as molecular tumour boards [144, 145], which bring together bioinformaticians, biologists and clinicians, can also help address the issue of translating complex data to be relevant to clinical care providers and patients.

The associated algorithmic approaches can require significant computational power. The resources offered by high-performance computing (HPC) can thus be exploited by bioinformaticians/computer scientists. There is now a major focus on the development of computing tools [146], platforms [147–149], data governance and infrastructure guidelines. A range of HPC solutions to support HTS is examined in the next section.

## HPC solutions

**Figure 3.**
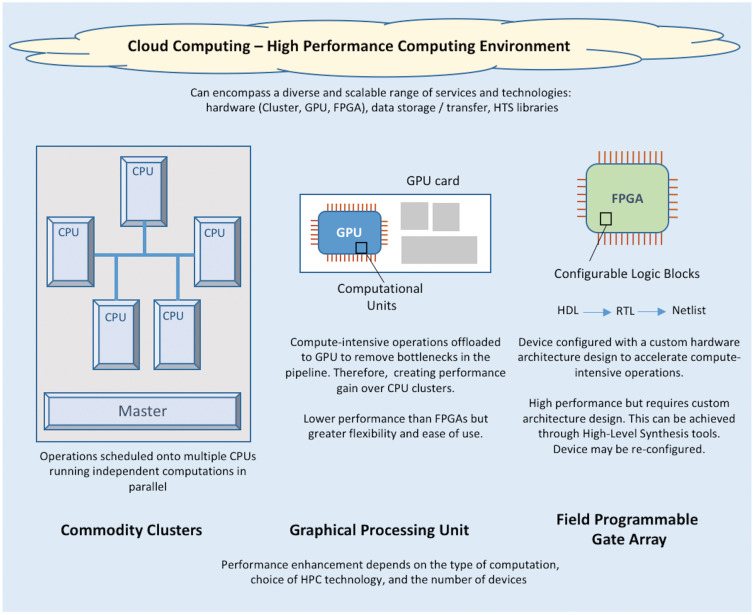
Overview of the difference options for high HPC. This is an illustration of commodity clusters, GPUs, FPGAs and cloud solutions. It highlights differences in performance, flexibility and level of custom design. *Note:* HDL, hardware description language; RTL, register-transfer level.

HPC can be achieved by using both hardware and software to partition tasks into groups of discrete and independent computations allowing them to be scheduled in parallel, with the seamless integration of results. There are a number of possible HPC solutions that can be tailored to meet computational demands. A short introduction is provided on these distinct HPC areas: cluster [150], graphics processing units (GPUs), cloud computing platforms [151] and field-programmable gate arrays (FPGAs) (Figure 3), together with example solutions in the HTS domain. Each approach differs in terms of technology, cost, performance, scalability and ease of implementation.

### Commodity clusters

Commodity clusters (Figure 3, Supplementary Table S1) have attained popularity within bioinformatics, because of their relatively low cost and scalability [152, 153]. They consist of regular desktops, with central processing units (CPUs) (for handling computations) or networked with servers (larger versions of desktops), [154] linked together to form a distributed computer system. This type of infrastructure enables parallel computing to be undertaken in (small) laboratories using low-cost hardware and standard software. However, technical experience is required in-house for the set-up; interconnection of desktops, set-up of the operating system and configuration of parallel programming software.

Open-source software frameworks such as Apache Hadoop [155] can support the scheduling of parallel operations, along with computational load and fault management. Hadoop [156] uses the MapReduce parallel programming framework, as popularized by Google, to facilitate the processing on data sets within the cluster infrastructure.

Kawalia *et al.* [157] describe a WES workflow, which incorporates MapReduce-like components for parallel calculations on clusters, enabling a ‘catch-up’ between data production and data processing and analysis (Table 1). MapReduce concepts have also been implemented in many other parallel solutions (Table 1) such as the Genome Analysis Toolkit (GATK) [146], a platform used for DNA- and RNA-Seq analysis in TCGA [42] and the 100 000 Genomes Project [158].

**Table 1.** Example HTS applications using cluster, GPU, cloud and FPGA HPC solutions

HPC solution	HTS personalized medicine applications
Cluster	Exome analysis workflow: [157] GATK [146] used by TCGA [42] and the 100 000 Genomes Project [158] Sequence alignment BLAST [159] Dimensionality reduction, Self-Organizing Maps (SOM) [160]
GPU	Process-intensive tasks such as RNA-seq alignment [161] and assembly [162]. Review of GPUs applied to RNA-Seq on cancer [163] such as parallel construction of Fuzzy C-Means clustering algorithm [164] Read mapping [165] Error correction [166]
Cloud	HTS read mapping algorithms such as CloudBurst [130], CloVR [149] and the Crossbow [167] Tailored bioinformatics platforms: BIOVIA ScienceCloud [147], DNAnexus [148], BaseSpace Sequence Hub [168] and Seven Bridges [169] Key projects have used public and private clouds, namely, International Cancer Genome Consortium and 100 000 Genomes Project
FPGA	Survey of FPGAs used in computational biology contexts: [170] General overview: [171, 172] Alignment algorithms: [173, 174]. Basic Local Alignment Search Tool (BLAST) FPGA accelerators: [175–177] Short read mapping: [174] Genome sequencing: MapReduce framework with acceleration on FPGA [178] Large-scale protein sequence alignment: [173] Complexity analysis of sequence tracts algorithm for low-complexity regions (LCRs) in protein sequences: [179] DRAGEN (Dynamic Read Analysis for Genomics) Processor**:** [180]

### GPU computing

GPUs (Figure 3, Supplementary Table S2) are card-based devices, which can be slotted into the graphics port of a laptop or desktop. One GPU card can comprise hundreds of computational units, in comparison with a CPU, offering increased scalability and processing performance [154, 181, 182]. Considering the price to performance ratio, parallel GPUs are potentially a more affordable and efficient option when compared with multiple, sequential CPUs [181–183].

Owing to the low cost and high-throughput processing capabilities, the GPU solution would be suitable for use in small research groups/laboratories. Code developed to run on CPUs cannot be ported to GPUs because of differences in architecture design. Therefore, computational expertise is a must. Also, data transfer between CPU and GPU memories [184] can create computational bottlenecks, limiting the potential for performance gain. Furthermore, modern GPUs have a complex architecture, which is vendor-dependent, e.g. Advanced Micro Devices Inc (AMD)\ATI Technologies Inc (ATI) or NVIDIA. Compute Unified Device Architecture (CUDA) [185] offered by NVIDIA is the most used platform and model for GPU parallel programming.

A large number of CUDA-compatible HTS data processing and analysis tools have been developed in the past for use with RNA-seq [163] and DNA-seq, e.g. Cushaw [186], BarraCUDA [187], SOAP3 [188], CUDASW++ [189] and SeqNFind [183], with a focus on sequence alignment using GPUs [186, 187] or CPUs and GPUs combined [189] (Table 1).

### Cloud computing

Cluster and GPU-based solutions can be implemented in-house. Cloud computing (Figure 3, Supplementary Table S3) refers to the use of off-site (remote) computers or servers for storage and processing, accessed by a user across a network connection. A major advantage of cloud solutions is that they provide adaptable storage and performance, without the necessity to deploy and maintain internal resources [147, 148], thereby providing scalable solutions to individual researchers through to large-scale clinical labs.

At the start of the ‘Big Data’ era, cloud computing was dominated by the use of Hadoop-based clusters. Since then, there has been a significant growth in the services provided by cloud vendors, offering data and project management tools that facilitate collaborations, regulate access to shared data and enable visualization and analysis of that data. Commercial options provide powerful solutions; however, organizations can develop their own private clouds using open-source facilities. These in-house servers may be regarded as more suitable solutions for sensitive data (e.g. patient information). Public clouds can be a viable option if sensitive data are encrypted, anonymized or used at a sufficiently abstract level omitting sensitive details [190].

The major players in commercial cloud provision, Amazon Web Services (AWS) Elastic Compute Cloud [191], Google Genomics [192] and Microsoft Azure [193], guarantee data security, with scalability and speed. High-profile HTS studies such as the 100 000 Genome Sequencing project have used private clouds while partnering with private companies, including AWS [194] and UK Cloud [195].

While commercial cloud solutions provide user-friendly interfaces with extensive toolkits, there are inherent disadvantages, including a lack of flexibility [196]. Open-source alternatives include platforms and pipelines, such as the alignment tool CloudBurst [130], a platform that combines virtual machine and cloud technologies, and CloVR [149] and the automated pipeline, Crossbow [167] (Table 1). However, open-source solutions arguably require more investment from the user, including system installation and management and the implementation of data analysis pipelines [196], all requiring substantial technical skills [149, 197].

### FPGA-based platforms

FPGA devices (Figure 3, Supplementary Table S4) are programmable integrated circuits, which consist of an array of configurable logic blocks each comprising local memory and computational units. The FPGA’s strength lies in its ability to reconfigure the dedicated hardware resources to meet the specific design needs of the implemented algorithms. FPGAs can yield great performance gains over GPUs for highly regular parallel operations. However, they are significantly more difficult to program, although this process has been simplified through recent high-level synthesis tools [198–200].

Furthermore, as with GPUs, FPGAs need to be part of a larger HPC environment for controlling which operations are sent to the device. However, vendors such as Intel have been developing hybrid CPU-FPGA Programmable Acceleration Cards [201] providing support for an acceleration stack of software, firmware and tools to assist this process. Recently, FPGAs have also found application in cloud platforms, such as Microsoft Azure [202] and Amazon AWS [203] providing additional flexibility and performance. Development would still need to be undertaken by bioinformaticians/computer scientists with computational skills in hardware design; however, the tools and solutions are evolving to make FPGA acceleration a more accessible option [199–203].

FPGAs have been used in computational biology settings [170], though to a lesser extent than cluster, GPU and cloud-based options with respect to HTS (Table 1). The most high-profile example involves Edico Genome, developers of the FPGA-powered DRAGEN Bio-IT Platform [4] and their partnership with Genomics England [125]. FPGAs are central to enabling this work, offering acceleration on sequencing pipeline computational bottlenecks, e.g. alignment and mapping. Owing to its high level of parallelism, DRAGEN can process a ‘whole human genome at 30x coverage in about 20 minutes, compared to 20-30 hours using a CPU-based system’ [4].

Each technology discussed offers advantages in their own right, providing performance gains dependent on the approaches taken. However, these solutions differ in terms of scalability, flexibility, cost and computational expertise for implementation. These solutions do not necessarily need to be taken individually, and the combination of clusters, GPUs, FPGAs and cloud-based workflows offers great promise to provide tailored genomic analysis solutions.

## Data management and governance

While technological and bioinformatics developments have paved the way for the generation of HTS data on smaller machines within reduced time frames and limited budgets, new challenges have arisen. Governance comes to the fore when considering the storage, sharing and privacy of the resultant data generated.

### Data size

While HTS data production costs are falling, the associated storage costs are reducing at a much slower rate [5]. Obtaining the actual sequence is only one part of a more complex overhead. Data storage, transmission, navigation and searches and the associated data processing resource and tools must also be considered [14, 204].

Management of large genomic data sets is discussed by Batley and Edwards [205]. Although data volume reduces from terabytes/gigabytes at the raw sequence stage, to gigabytes/megabytes once stored in text sequence format, there are further challenges in terms of data searchability and accessibility. Using standard sequence comparison algorithms is time-consuming; furthermore, tools such as BLAST are computationally intensive [160, 177, 206].

Compression techniques offer another effective storage solution [207–209], often comparing sequences against reference genomes [204, 210]. In Brandon *et al.* [204] resultant differences were encoded using entropy-based methods such as Huffman. Through such techniques, a 345-fold compression rate was achieved, in one example reducing a 56 MB sequence down to 167 KB.

The graphical representation and interpretation of data is also an important factor, particularly as data sets increase in size and diversity, leading to the development of visualization tools [211, 212].

### Data security

Genetic information can provide the ultimate insight into the health of private individuals. As such it needs to be treated with the greatest levels of confidence, security and ethical standards. Once such data become a component of a computer infrastructure, high-level cyber security measures need to be used, namely, encryption, authentication and authorization [213]. Furthermore, before inclusion in a publicly available HTS repository, donor anonymity must be safeguarded [214, 215].

The US Presidential Commission for the Study of Bioethical Issues recommended that there needs to be ‘strong baseline protections while promoting data access and sharing’ [216]. Such sharing should be with the goal of progressing biological knowledge for public benefit.

Recognizing the translational challenges posed by data repositories [217], bodies such as the Electronic MEdical Records and GEnomics (eMERGE) Consortium [218] have contributed towards developing good practice guidelines and standards in the governance of genomic data (Table 2). In sharing or publishing data, ensuring the anonymity of patients is routinely achieved through de-identification. In certain research areas, e.g. the study of rare diseases, there is a risk of traceability through publication of associated information such as age, ethnicity and gender [214]. The security and storage of such data can be further protected by considering it as protected health information (PHI).

**Table 2.** US and EU organizations established for the protection of health and personal data

Legislation	Date	Description
Health Insurance Portability and Accountability Act (HIPPA)	1996	HIPPA safeguards individuals’ PHI [219]. Its privacy rules set guidelines on how health data can be disseminated through suitable de-identification. Two standards (Safe Harbor and Expert Determination) may be used for the de-identification process [220]
Health Information Technology for Economic and Clinical Health Act (HITECH)	2009	HIPAA was later supplemented by the Health Information Technology for Economic and Clinical Health Act (HITECH)
Genetic Information Nondiscrimination Act of 2008 (GINA)	2008	A US Federal Law that prohibits discrimination in health insurance and employment as a result of genetic information [221]
		Note however that GINA does not provide complete coverage, e.g. it does not prohibit health insurers from using genetic information in determining insurance premiums
Patient Protection and Affordable Care Act (ACA) of 2010	2010	Makes it illegal for health insurers to raise premiums or remove cover for those with pre-existing conditions
Directive 95/46/EC of the European Parliament and Council of the European Union (EU)	1995	This directive covers the protection of individuals with regard to the processing of personal data and on the free movement of such data. (Official Journal of the European Union L 281: 0031–0050.)
Directive (EU) 2016/680	With effect from 2018	Directive 95/46/EC will be repealed and replaced by the regulation and directive on the protection of natural persons with regard to the processing of personal data—General Data Protection Regulation (GDPR) [222, 223]
Regulation (EU) 2016/679/		

Despite the computational benefits of cloud-based solutions, the security of data and subsequent analysis held within such frameworks are still considered bottlenecks [224]. Cloud-based providers have responded, through the development of in-built facilities, such as encryption, auditing, data backup and recovery, to comply with data governance and management regulations as required, e.g. by the Health Insurance Portability and Accountability Act (HIPAA) of 1996 [225]. Examples include AWS DNAnexus [148] and the hybrid offering from Microsoft Azure [226]. HTS-tailored alternatives such as BC Platforms can be implemented in-house, targeting security- and cost-conscious end users [227].

Genetic databases can be shared successfully and at a global scale. One such example, GenBank, is a generic sequence database (nucleotide sequences and their protein translations) established and coordinated by the National Center for Biotechnology (part of the National Institutes of Health in the United States) [228]. The initiative is part of the International Nucleotide Sequence Database Collaboration (INSDC), comprising members from DNA DataBank of Japan (DDBJ) and the European Nucleotide Archive (ENA) [229–231].

The INSDC collections, comprising submissions from both small- and large-scale independent laboratories, are freely available. INSDC adds to its database daily, with a GenBank public update every 2 months. The expansion of GenBank’s database has doubled approximately every 18 months [232], highlighting the growth, supporting infrastructure and acceptance, in sharing sequencing data. The European Bioinformatics Institute (EBI) repository, ArrayExpress [233], also allows researchers to upload their HTS data sets for public distribution. In depositing data, standardization is required, e.g. Minimum Information About a Microarray Experiment (MIAME) and Minimum Information about a high-throughput nucleotide SEQuencing Experiment (MINSEQE) guidelines [234].

## Ethical issues surrounding genetic data

There are multiple ethical challenges when handling HTS data. Apart from the obvious examples, e.g. a data breach, there are other more subtle, unanticipated incidences. A key case is that of Henrietta Lacks. Henrietta died from cervical cancer in 1951; yet, the cell line derived from her tumour (HeLa) is still replicating and has become a pivotal resource as a preclinical model [235]. The ethical conflict in this example arose from the lack of consideration for the family of Lacks and indeed lack of consent with regard to the publication of the results from sequencing of the cell line. An uploaded sequenced HeLa sample was retracted from the ENA because of privacy concerns in 2013 [236, 237]. This highlighted the lack of clarity and legislation surrounding ownership over donated samples and the potential impact for family members.

Furthermore, if we consider the process of genetic discovery, what is the line between research and clinical diagnosis [238]? This presents many quandaries for retrospective research projects in particular. If a cancer patient, who had agreed to donate material from their tumour for a research project, was found to possess a particular gene mutation, do researchers have a responsibility to inform the patient and/or the patient’s family [75]? If a compound had not yet been approved for treating this particular mutation, this new knowledge could not be used to advance the health of the patient.

If we take it on ourselves to sequence our DNA, could this impact insurance? Table 2 provides a summary of current legislation for the United States and European Union (EU). Both the US Health Information Technology and Clinical Health Act (HITECH) and the EU Directive 2016/680 Regulation 2016/679 provide safeguarding of individuals health data and how it is handled and transmitted. As part of the 2010 US Patient Protection and Affordable Care Act (ACA) cancer risk assessment, via genetic testing, was promoted as a preventive measure under the assurance that no person would be negatively impacted by changes in cost or provision in their insurance cover [239]. While the legislation was not fully comprehensive of all conditions, the ‘good-will’ of preventive medicine, based on personalized risk, was present. However, with current changes in US legislation and the development of the ‘Preserving Employee Wellness Programs Act’ [240], concerns have been raised regarding the protection of employees’ rights [241]. The full implications are unclear, but it does appear that employees will be given fewer options in terms of privacy, contradicting the legislation as set out by the 2008 Genetic Information Nondiscrimination Act (GINA). If employers are empowered to this extent, there is a risk that the public will lose confidence in, and acceptance of, genetic testing, impacting negatively on the uptake in preventative screening.

## Discussion

We have provided a broad overview of the facilitators and barriers associated with the widespread adoption of HTS in personalized medicine (Figure 2). Technological advances have been a key driver in offering affordable and efficient access to sequencing solutions, with the 1 h genome sequence now a reality [3] and Illumina forecasting a $100 cost per genome within 3–10 years [242]. Illumina have also been developing chip-based sequencing incorporating their DNA- and RNA-Seq technologies into a semiconductor device with the resulting product launched in 2017 [243].

In terms of computational power to perform analysis, technology is at a significant stage. Cloud platforms offer scalability, security and computational performance [148, 191, 193, 227, 244]. Meanwhile, advances beyond the cloud also continue with visions for silicon chip-based and mobile solutions [243, 245] with an eye towards real-time processing of HTS data.

A multidisciplinary approach to technological development and translational research is required to promote HTS within personalized medicine. However, barriers must be acknowledged; the phenomenal production rate of sequencing data has the potential to overwhelm current computing infrastructures and bioinformatics resources [5, 14, 246].

Addressing heterogeneity in output through standardization is crucial when considering HTS data integration with healthcare informatics structures. In particular, to consider assimilation, electronic healthcare records, raw HTS data and associated ontologies, must be normalized [247]. This challenge has been recognized with a call for replicable and auditable workflows [171]. This must be supported by an investment in informatics infrastructure, with a focus on storage and software development [248]. Patient consent highlights the need for a goodwill ‘buy-in’ by the general public, in terms of data-sharing, alongside a closer patient involvement [215]. This can only be achieved if there is confidence in privacy assurances.

A disconnect between HTS data production and the analytics required to facilitate biological understanding still exists. Li *et al.* [249] acknowledge that ‘integrative analysis of this rich clinical, pathological, molecular and imaging data represents one of the greatest bottlenecks in biomarker discovery research in cancer and other diseases’. This may be addressed by larger studies such as the 100 000 Genome Project, which aims to sequence the genomes of 100 000 patients enabling downstream integration of results with associated clinico-pathological data [158] or the PatientsLikeMe project, which is collaborating with governmental and pharmaceutical companies [250].

Such large-scale projects will depend on the standardization of data management and analysis, if HTS-produced biomarkers are to be translated into the clinic for patient diagnosis and treatment stratification. Also, avoiding the ‘Winner’s Curse’ can be achieved through the use of appropriate study design, robust statistical methods and validation [251, 252]. Standardized, replicable pipelines, from sequencing to downstream analysis, are therefore now required, such as the FDA/HUPO Proteomics Standards Initiative-established Sequencing Quality Control (SEQC) project [253]. While we are still playing bioinformatics catch-up with the HTS wave, new small-scale real-time sequencing solutions are coming on-stream [3, 4, 32]. It is essential that we apply the lessons learned from the previous computational and governance challenges to keep pace with new HTS developments.

## Conclusion

To ensure its place within the personalized medicine arsenal, first and foremost, the computational resources required for HTS processing must be accessible in terms of costs, skills and efficiency. Standardization in HTS processing and analytical pipelines will facilitate validation and ensure replication of results, within clinically relevant time frames. This, in turn, alongside multidisciplinary collaboration, will enable its full integration into patient care and treatment, through the provision of new diagnostic, predictive and prognostic tests.

**Key Points**

- An overview on sequencing technologies and their role in personalized medicine.
- Identification of current bottlenecks in the translation of ‘omic’ data to personalized medicine.
- Up-to-date review on current computational technologies, infrastructure and future solutions to handling and analysing of sequencing data in real time.
- The changing required in clinical governance in the face of rapid adoption of sequencing technologies into clinical workflows.
- This paper provides a review of high-throughput sequencing in the context of biomedical research to clinical use with a focus on applications, pipelines, processes and technologies along with challenges.

## Supplementary Data

Supplementary data are available online at https://academic.oup.com/bib.

## Supplementary Material

bby051_Supp

## Notes

**Gaye Lightbody** is a Lecturer in Computing Science. Her research career has spanned areas of high-performance hardware architectures for signal and data processing systems, biosignal analysis and artificial intelligence.

## Notes

**Valeriia Haberland** is a Senior Research Associate (PhD in Computer Science). Her research interests included high-performance computing and automated data matching. Currently, she is interested in causal inference and text mining.

## Notes

**Fiona Browne** is a Lecturer in Computing Science. Her research interests focus on artificial intelligence and information fusion in multidisciplinary fields including Bioinformatics.

## Notes

**Laura Taggart** is a Senior Scientist with a focus on genomics in the context of stratifying cancer patients with the aim of improving therapeutic outcomes for patients as well as a current interest in the development of liquid biopsies.

## Notes

**Huiru Zheng** is a Professor of Computer Science. Her research interests include integrative data analytics in the field of systems biology and systems medicine.

## Notes

**Eileen Parkes** is an Academic Clinical Lecturer in Medical Oncology. Her PhD led her from DNA damage to immunobiology, and she continues exploring this rich interface in translational and clinical research.

## Notes

**Jaine K. Blayney** is a Lecturer in Translational Bioinformatics with research interests in data integration through compositional statistics approaches, and the development of disease-agnostic bioinformatics and biostatistics techniques to inform biomarker discovery and validation.
