# Supplementary material for: Review of applications of high-throughput sequencing in personalized medicine: barriers and facilitators of future progress in research and clinical application
Source: Brief Bioinform. 2019 Jun 14;20(5):1795–811. doi: 10.1093/bib/bby051 (PMC6917217; doi:10.1093/bib/bby051)
Supplement: bby051_Supp [file bby051_supp.docx]

**Supplementary**

This section provides supplementary technical information about each of the four HPC areas discussed in the main body of the paper, namely, Cluster (***Supplementary Table 1***), GPU (***Supplementary Table 2***), Cloud (***Supplementary Table 3***), and FPGA solutions (***Supplementary Table 4***).

Supplementary Table 1: Commodity Clusters High Performance Computing Solutions – Technical Detail

| **Cluster HPC Solution** | |
| --- | --- |
| **Advantages / Disadvantages** | |
| **Advantages** (+) Low-cost setup, scalable, Fault tolerance  **Disadvantages** (-) Setup and maintenance requires computational experience in both hardware and software | |
| **Hardware:** | |
| Standard desktops/servers with multiple processing cores | Desktops interconnected via a local area network. Operating system and processing software installed to enable parallel processing |
| **Software:** | |
| a) Apache Hadoop  [1] | Enables the computations to be handled in parallel through sorting the input data into independent slices scheduling them to be processed in parallel without conflict. The framework aims to balance the computational load. |
| b) MapReduce | Employed by Hadoop and popularised by Google [2]. MapReduce recognised for its fault tolerance. This framework consists of *Map* and *Reduce* stages, where input data is first split and presented as intermediate key-value pairs (mapping). The pairs are sorted by their keys and the values aggregated under their respective keys at the assigned reducer nodes [3]. The values are then processed for each key (reducing), e.g. counting name frequency. |
| **c)** Message Passing Interface (MPI)  [4] | Often compared to the MapReduce paradigm in terms of flexibility [5] and fault tolerance. Applications which use MPI can create check-points in order to improve their own fault tolerance. Generally, MapReduce is considered to be a more suitable solution for data-independent tasks, as MPI allows more control over data communication. The use of these paradigms is not mutually exclusive, as there are libraries which consider both, such as MR-MPI [6]. |
| d) SeqPig  [7] | An Hadoop-based set of HTS data manipulation tools based on Apache Pig. This toolkit can be used for shared- or distributed-memory systems which enhances its application in different types of clusters. Parallel versions of well-known algorithms such as BLAST (basic local alignment search tool), to identify regions of sequence similarity, and SOM (self-organizing maps), a version of artificial neural networks used in dimension reduction, have also been developed using this approach [8]. |
| e) Apache Spark | Spark’s efficiency can be attributed substantially to the usage of Resilient Distributed Datasets (RDDs) which represents an innovative in-memory storage abstraction, as proposed by Zaharia et al. [9]. It is argued that this memory abstraction can be more efficient for a wider range of applications requiring data reuse, than other related, but specialised, solutions (e.g. HaLoop [10], Pregel [11]). The main advantages of RDDs lie in their applicability to more general cases of data reuse (including interactive applications) and efficient fault-tolerance solutions [9]. The latter is achieved by recording a “lineage” of the data (RDD), i.e. the operators (e.g. “map”) that were applied to produce the dataset, which then can be used to reproduce this dataset (or its partition) from other RDDs if necessary. This approach substantially reduces communication and storage costs compared to earlier solutions, which replicated the data (or recorded the updates) across commodity computers [9]. |

Supplementary Table 2: Graphical Processing Units High Performance Computing Solutions – Technical Detail

| **GPU HPC Solution** | |
| --- | --- |
| **Advantages / Disadvantages** | |
| **Advantages** (+) Low-cost setup, scalable, Fault tolerance  **Disadvantages** (-) Setup and maintenance requires computational experience in both hardware and software | |
| **Hardware:** | |
| A parallel co-processor present in common desktops used to render graphics | A GPU consists of high volume of programmable units. These units can execution thousands of threads simultaneous making them advantageous to parallel tasks such as sequence alignment. GPUs also have a memory hierarchy [12], which can be classified into the types of memory privately used by the respective streams (threads); shared or read-only memories used within a multiprocessor, and a global device memory used by all multiprocessors. A global memory is also used to exchange data with the CPU memory  Example vendor: NVIDIA. |
| **Software:** | |
| NVIDIA, Compute Unified Device Architecture (CUDA) | CUDA is a well-established architecture for GPU programming and the most commonly applied in the Bioinformatics domain [13]. CUDA can only be used on NVIDIA cards in comparison to OpenCL. |
| Open Computing Language (OpenCL) | Open Computing Language (OpenCL) [14] is a viable alternative solution to CUDA, being an open programming standard for parallel applications that is not dependent on the hardware, and hence regarded as “truly open and royalty-free” [15]. Some well-known GPU vendors, i.e. NVIDIA and AMD, have targeted their implementations of OpenCL towards their respective GPUs [16]. However, OpenCL, is reportedly harder to program than alternatives [14] and solutions ported to OpenCL may be less efficient [17]. |
| Other environments and tools | With regard to statistical data analysis and visualisation, the open-source R-environment is being increasingly used by bioinformaticians [18] to accelerate computational applications, e.g. the CPU-based Simple Network of Workstations (SNOW) [19], the GPU-based gputools [20], and permGPU [21]. |

Supplementary Table 3: Cloud High Performance Computing Solutions – Technical Detail

| **Cloud HPC Solution** | |
| --- | --- |
| **Advantages / Disadvantages** | |
| **Advantages** (+) On demand access to high performance computational servers at a low-cost point.  Server technical support not required. Running costs of servers not incurred.  **Disadvantages** (-) Privacy and legality around the analysis of sensitive information on a privately-owned server in terms of ownership | |
| **Hardware / Software:** | |
| Availability of servers for processing, storage of a range of computational tasks available on demand via the internet. | Prominent cloud venders include Amazon, Microsoft and Google.  Clouds have been widely used in bioinformatics [22,23]with vendors providing computing infrastructure, data storage and processing and sharing massive amounts of data as a service on a “pay as you go” basis, using a Service Level Agreement (SLA) protocol. Clouds allow users to not only use CPU capacity of their resources, but some also offer GPU or even FPGA [24–26] acceleration. An example of this is the Amazon Elastic Compute Cloud (EC2) instance type, G2.  Important considerations are required relating to data control and ownership, commercialization and conflicts of interest when using private clouds. |
| Hybrid clouds | Hybrid clouds, such as those provided by Microsoft [27], IBM [28], Cisco [29], and Amazon [30], combine the advantages of in-house solutions and public clouds, with the aim of being both cost and resource efficient [31]. However, the cost of hybrid solutions may increase for more data-intensive applications, especially, those which require extensive communication between public and in-house clouds. In such cases, a larger deployment of an in-house solution would be preferable. |
| Services and costs | There is an evident shift of research focus from a single data centre approach to that of larger and more complex clouds, denoting a move towards a vision of an *InterCloud* [32]*,* where smaller clouds provided by different vendors are combined into a cloud of clouds. InterCloud also raises a new set of issues, such as the standardization of security policies, scheduling complexities and the sharing of workloads among sub-clouds.  Clouds are usually commercial with a “pay-as-you-go” pricing structure. However, there are still no clear standards regarding cloud pricing models, which should ideally account for variations in a user’s quality of service [32].  When data is to be stored in a globally distributed manner, the cost of data transfer will be in direct correlation to the user’s geographical location and the size of dataset in question [33]. Hence, costs will increase substantially for computationally- and data- intensive tasks. Here, “cost” refers to the monetary payments per service usage [33], while “cost” in the context of grids usually refers to the efficient processing of tasks, e.g. avoiding long queues, and slow data transfer [34]. However, while cloud users are concerned initially with the financial costs, processing costs, such as data acquisition and task execution, are becoming increasingly important. Intuitive solutions would include using the nearest data centre or moving a task closer to the data. However, constraints still exist, e.g. data transfer from a non-local site, involved in the “global” cloud [34]. Pandey et al. [33] focus on minimizing the communication and computation cost in clouds for data-intensive tasks when using non-linear optimization methods. Computation, network and data transfer costs are considered in the Data Intensive and Network Aware (DIANA) scheduling for grids [34,35]. Others, e.g. Network-Aware Cost-Efficient Resource allocation (NACER) for clouds [36], focus on the communication costs specifically.  Aside from task execution costs, what are the costs involved in the provision and maintenance of the data centres themselves? Greenberg et al. [37] estimate that servers account for the largest proportion of costs, with up to tens of thousands of items in each centre. Power consumption, network connections, etc., also contribute significantly to costs. The authors emphasise the benefits and challenges of a geographically diverse placement of data centres. On the one hand, a “geo-diverse” distribution of data centres may reduce the response latency and communication cost for the end users, on the other hand, this cost would depend on whether users’ applications are run on one or multiple data centres, and in the latter case, on the distance between those centres. |

Supplementary Table 4: Field Programmable Gate Arrays High Performance Computing Solutions – Technical Detail

| **FPGA HPC Solution** | |
| --- | --- |
| **Advantages / Disadvantages** | |
| **Advantages** (+) Speed of processing through configuration of process specific tasks performed on dedicated hardware without the overheads of computational processing.  **Disadvantages** (-) Can be complicated to code/debug circuit design requiring expertise in this area | |
| **Hardware:** | |
| In general devices consist of configurable logic blocks consisting computational arithmetic units with localized memory. | Configurable devices on which a tailored hardware design can be implemented.  The design process is more complex requiring an architecture design for synthesis onto a FPGA device. [38].  Example vendors include Xilinx [39] and Altera, (now owned by Intel) [40].  Traditionally design flow needs to translate the design from the *behavioural* representation (often modelled in c) through to the *structural* representation (using Hardware description language such as VHDL or Verilog), then onto the *physical* representation (synthesized netlist placed and routed on the device). Optimum design requires expertise as each of these stages. |
| **Software:** | |
| Vivado [41] by Xilinx  Altera offer an approach based on OpenCL [42] | Progress has been made in the area of High-Level Synthesis which raises the abstraction of hardware design to be more in line with software approaches (see chapter 7 of [38]). Tools such as Vivado [41] by Xilinx allow c-based design. While Altera offer an approach based on OpenCL [42]. Both of these have the potential to be a game changer for FPGA uptake and the purchase of Altera by Intel [40] plus the adoption of FPGA solutions in the cloud surely indicate as such [24–26]. |

**Supplementary References**

1. The Apache Software Foundation. Welcome to ApacheTM Hadoop®! 2014; http://hadoop.apache.org/.

2. Dean J, Ghemawat S. MapReduce: Simplified data processing on large clusters. Sixth Symp. Oper. Syst. Des. Implement. 2004; 51:107–113

3. Taylor RC. An overview of the Hadoop/MapReduce/HBase framework and its current applications in bioinformatics. BMC Bioinformatics 2010; 11 Suppl 1:S1

4. Barney B. Message Passing Interface (MPI). 2017; https://computing.llnl.gov/tutorials/mpi/.

5. Chen WY, Song Y, Bai H, et al. Parallel spectral clustering in distributed systems. IEEE Trans. Pattern Anal. Mach. Intell. 2011; 33:568–586

6. Plimpton SJ, Devine KD. MapReduce in MPI for Large-scale graph algorithms. Parallel Comput. 2011; 37:610–632

7. Schumacher A, Pireddu L, Niemenmaa M, et al. SeqPig: Simple and scalable scripting for large sequencing data sets in hadoop. Bioinformatics 2014; 30:119–120

8. Symp. Parallel Distrib. Process. Work. PhD Forum 2011; 481–489

9. Zaharia M, Chowdhury M, Das T, et al. Resilient distributed datasets: A fault-tolerant abstraction for in-memory cluster computing. 9th USENIX Conf. Networked Syst. Des. Implement. 2012; 2–2

10. Bu Y, Howe B, Balazinska M, et al. HaLoop. Proc. VLDB Endow. 2010; 3:285–296

11. Malewicz G, Austern MH, Bik AJC, et al. Pregel: a system for large-scale graph processing. Proc. 2010 Int. Conf. Manag. data - SIGMOD ’10 2010; 135

12. Fei X, Li K, Yang W, et al. CPU-GPU Computing: Overview, Optimization, and Applications. Innov. Res. Appl. Next-Generation High Perform. Comput. 2016; 159–193

13. Shi H, Schmidt B, Liu W, et al. A Parallel Algorithm for Error Correction in High-Throughput Short-Read Data on CUDA-Enabled Graphics Hardware. J. Comput. Biol. 2010; 17:603–615

14. Nobile MS, Cazzaniga P, Tangherloni A, et al. Graphics processing units in bioinformatics, computational biology and systems biology. Brief. Bioinform. 2016; 182:bbw058

15. AMD - Developer Central. The AMD OpenCLTM Zone – Accelerate Your Application. 2017; http://developer.amd.com/tools-and-sdks/opencl-zone/.

16. Stone JE, Gohara D, Shi G. OpenCL: A parallel programming standard for heterogeneous computing systems. Comput. Sci. Eng. 2010; 12:66–72

17. Harvey MJ, De Fabritiis G. Swan: A tool for porting CUDA programs to OpenCL. Comput. Phys. Commun. 2011; 182:1093–1099

18. R Foundation. R: The R Project for Statistical Computing. 2017; https://www.r-project.org/.

19. Tierney L. Simple Network of Workstations for R, Department of Statistics and Actuarial Science University of Iowa. 2017; http://homepage.stat.uiowa.edu/~luke/R/cluster/cluster.html.

20. Buckner J, Wilson J, Seligman M, et al. The gputools package enables GPU computing in R. Bioinformatics 2009; 26:134–135

21. Shterev ID, Jung S-H, George SL, et al. permGPU: Using graphics processing units in RNA microarray association studies. BMC Bioinformatics 2010; 11:329

22. Langmead B, Nellore A. Cloud computing for genomic data analysis and collaboration. Nat. Rev. Genet. 2018; 19:208–219

23. Hashem IAT, Yaqoob I, Anuar NB, et al. The rise of ‘big data’ on cloud computing: Review and open research issues. Inf. Syst. 2015; 47:98–115

24. Fieldman M. Microsoft Goes All in for FPGAs to Build Out AI Cloud | TOP500 Supercomputer Sites. Top 500 2016; https://www.top500.org/news/microsoft-goes-all-in-for-fpgas-to-build-out-cloud-based-ai/.

25. Xilinx. Accelerated Cloud Services. 2018; https://www.xilinx.com/products/design-tools/cloud-based-acceleration.html.

26. AWS. Amazon EC2 F1 Instances - Run Customizable FPGAs in the AWS Cloud. 2018; https://aws.amazon.com/ec2/instance-types/f1/.

27. Microsoft. Hybrid Cloud Solutions | Microsoft. 2018; https://www.microsoft.com/en-gb/cloud-platform/hybrid-cloud.

28. IBM. IBM Cloud Computing: IBM Cloud Private - United Kingdom. 2018; https://www.ibm.com/cloud-computing/uk/en/private-cloud.html.

29. CISCO. Cisco Cloud. 2018; https://www.cisco.com/c/en_uk/solutions/cloud/overview.html.

30. AWS. AWS Storage Gateway – Hybrid Cloud Storage – Amazon Web Services (AWS). 2018; https://aws.amazon.com/storagegateway/.

31. Mazhelis O, Tyrvainen P. Role of Data Communications in Hybrid Cloud Costs. 2011 37th EUROMICRO Conf. Softw. Eng. Adv. Appl. 2011; 138–145

32. Buyya R, Pandey S, Vecchiola C. Cloudbus Toolkit for Market-Oriented Cloud Computing. 2009; 24–44

33. Pandey S, Barker A, Gupta KK, et al. Minimizing execution costs when using globally distributed Cloud services. Proc. - Int. Conf. Adv. Inf. Netw. Appl. AINA 2010; 222–229

34. McClatchey R, Anjum A, Stockinger H, et al. Data intensive and network aware (DIANA) grid scheduling. J. Grid Comput. 2007; 5:43–64

35. Anjum A, McClatchey R, Willers I. Data analysis with the DIANA meta-scheduling approach. J. Phys. Conf. Ser. 2008; 119:072004

36. Ahvar E, Ahvar S, Crespi N, et al. NACER: A Network-Aware Cost-Efficient Resource Allocation Method for Processing-Intensive Tasks in Distributed Clouds. 2015 IEEE 14th Int. Symp. Netw. Comput. Appl. 2015; 90–97

37. Greenberg A, Hamilton J, Maltz DA, et al. The Cost of a Cloud : Research Problems in Data Center Networks. ACM SIGCOMM Comput. Commun. Rev. 2009; 39:68–73

38. Woods R, McAllister J, Lightbody G, et al. FPGA-based implementation of signal processing systems. John Wiley & Sons, 2017;

39. Xilinx. FPGAs and 3D ICs. https://www.xilinx.com/products/silicon-devices/fpga.html.

40. Intel Altera. Accelerate the World | Intel® FPGAs, the multi-function Accelerator of Choice. 2018; https://www.altera.com/.

41. Xilinx. Xilinx: Vivado design suite. 2018; https://www.xilinx.com/products/design-tools/vivado.html.

42. Intel. Intel FPGA SDK for OpenCL - Overview. 2018; https://www.altera.com/products/design-software/embedded-software-developers/opencl/overview.html.
